# Factor Analysis of the Brazilian Questionnaire on Adherence to Ketogenic Dietary Therapy: Keto-Check

**DOI:** 10.3390/nu15173673

**Published:** 2023-08-22

**Authors:** Lenycia de Cassya Lopes Neri, Alexandre Archanjo Ferraro, Monica Guglielmetti, Simona Fiorini, Letícia Pereira de Brito Sampaio, Anna Tagliabue, Cinzia Ferraris

**Affiliations:** 1 Faculty of Medicine, Department of Pediatrics, University of Sao Paulo, São Paulo 05403-000, Brazil; ferraro@usp.br (A.A.F.);; 2 Ketogenic Metabolic Therapy Laboratory, Department of Public Health, Experimental and Forensic Medicine, University of Pavia, 27100 Pavia, Italy; 3 Laboratory of Food Education and Sport Nutrition, Department of Public Health, Experimental and Forensic Medicine, University of Pavia, 27100 Pavia, Italy

**Keywords:** adherence, compliance, ketogenic diet, drug-resistant epilepsy

## Abstract

Background: several strategies are used to assess adherence to ketogenic dietary therapies (KDTs), the most commonly used being ketonemia or ketonuria, despite their limitations. The purpose of this article is to carry out an exploratory and confirmatory factor analysis on the proposed Keto-check (adherence’s KDT Brazilian questionnaire). Methods: there was a methodological study of a quantitative nature, complementary to the analysis realized previously, with a complimentary sample. The factorial analysis was performed with Factor software for parallel exploratory analysis, replicability, and confirmatory factor analysis. Graphical representation was created according to the number of factors resulting from the analysis. Results: 116 questionnaires were reached by complementary data collection (*n* = 69 actual data, complementing *n* = 47 previous data) through online forms. A polychoric correlation matrix suitability analysis resulted in a significant Bartlett statistic (*p* = 0.0001) and a Kaiser–Meyer–Olkin (KMO) test of 0.56. The parallel factorial analysis resulted in two factors, graphically represented as “efficacy” and “adherence”. A confirmatory factor analysis, considered fair, indicated an RMSEA of 0.063, NNFI resulted in 0.872, CFI in 0.926, and GFI in 0.897. Conclusion: this study confirms the validity of Keto-check through a more detailed analysis. Adherence is the key to improving the effectiveness of KDTs; therefore, improving knowledge about it can lead to a better healthcare approach.

## 1. Introduction

Ketogenic dietary therapy (KDT) is an option for the treatment of drug-resistant epilepsy, with some syndromes having proven efficacy in more than 70% of cases with a reduction in frequency, duration, or intensity by 50% of the initial value [1]. More generally, ketogenic dietary therapies (KDTs) can provide potential therapeutic benefits in patients with neurological disorders [2,3,4,5,6,7] by effectively controlling the balance between pro- and antioxidant processes and proexcitatory and inhibitory neurotransmitters, and modulating inflammation or changing the composition of the gut microbiome [8]. Nowadays, various KDTs are available and used in the treatment of neurological disorders [9].

KDTs are rich in fat, adequate in protein, and restricted in carbohydrates, with currently four different approaches: classical ketogenic diet (CKD) [10], modified Atkins diet (MAD) [11], medium chain triglycerides (MCT) [12], low glycemic index (LGIT) [13], with the CKD being the most restrictive and less palatable, although the most effective in determining ketosis [8]. The choice of protocol by the healthcare team and caregivers should take into account individualization to improve adherence by the patient and family [1].

The World Health Organization (WHO) defines adherence as the degree to which a person’s behavior corresponds to the health professionals’ recommendations in relation to medication, following a diet, or modifying lifestyle habits [14]. Other authors, such as Fawcett and colleagues [15], report that the term “adherence” is not, as often verified in the scientific literature, synonymous with “compliance”. The term adherence would have a less paternalistic effect than compliance, with more active participation in the treatment process, as opposed to merely obeying the treatment [15].

Adherence to treatment with KDTs is often difficult for patients and caregivers [16,17,18,19,20,21]. Complaints of monotonous diet and low patient acceptance are recurrent in clinical practice. A meta-analysis of 11 KDT studies indicated a combined patient compliance of 45%. Adherence failure could be attributed to adverse effects of the diet, psychosocial factors, or the food restriction caused by the diet [22].

Adherence is a latent variable that is difficult to measure. Several studies try to quantify adherence through direct methods, such as blood [23] or urinary biomarkers [24], or indirect methods, for example, patients’ reports [25], clinical records, or questionnaires [26,27]. None of these methods is considered a gold standard [28] and the discrepancies between both types of measurements must be taken into consideration when it is necessary to assess the adherence to the treatment [29].

In the literature, to assess adherence to ketogenic therapies, several strategies are used. Commonly it is used by direct methods, such as measuring ketonemia (presence of β-hydroxybutyrate in the blood) or ketonuria (presence of acetoacetate in the urine). Both measurements do not correlate with each other and have their limitations [30,31].

In KDT studies, there is no consensus about compliance measuring methods. Each study proposes a different way of indirectly measuring adherence to treatment, some authors through the patients’ reports [32], through food records with weighted meals [33], motivations referred by the patients to start the diet [34], reports of expectations, and factors that influence the duration of the diet [35]. Just a few studies created an instrument to measure adherence [36,37,38]. In the United Kingdom, one study created a score based on the needs and concerns of patients [39]. Also, an American instrument was created to verify adherence to KDT compared to the Mediterranean diet, but in a controlled environment by providing food during the study [40].

A Brazilian questionnaire was recently published with the purpose of indirectly measuring adherence to the ketogenic diet of patients following KDT [36]. However, the article has some limitations; for example, it presented Cronbach’s coefficient as a measure of the instrument’s internal consistency and a factor analysis was not performed to verify the validity of the instrument. Currently, some articles already point out that the Cronbach coefficient is not the most appropriate method for checking internal consistency [41,42].

Factor analysis (FA) is one of the main tools in the development, evaluation, refinement, and use of psychometric tests. It can be defined as a set of statistical techniques used to define the underlying structure in an array of data. It evaluates how a certain number of items (observed by the instrument) can be grouped into a smaller number of latent variables, called factors, and thus explain their interrelationships [43].

The purpose of this article is to carry out an exploratory and confirmatory factor analysis on the previously published Keto-check data to understand how many and which factors are involved with the latent variable analyzed: adherence to ketogenic therapy.

## 2. Materials and Methods

This is a methodological study of a quantitative nature, complementary to the analysis realized in the article on the validation of the ketogenic diet adherence questionnaire named Keto-check [36]. This protocol has been approved by the Research Ethics Committee (CEP) of the Medical School of Universidade de São Paulo, with Certificate of Presentation for Ethical Appreciation (CAAE) 19812919.2.0000.0068 and approval n. 3.735.637. Written informed consent has been obtained from all patient(s) or caregivers to publish this paper.

The questionnaire consists of 10 statements with 5 Likert scale agreement alternatives to the exposed sentence (the complete questionnaire can be found in the Supplementary Material, Table S1). The presented sentences report the patient’s perception of improvement in quality of life, seizures reduction, monitoring ketosis, checking the weight of consumed food, keeping strictly into the prescribed diet, avoiding using drugs with carbohydrates, attendance in consultations, checking food labels, and cooking ketogenic recipes at home.

The present study was carried out with patients undergoing ketogenic therapy for refractory epilepsy at the Instituto da Criança of the Hospital das Clínicas of the University of São Paulo, Brazil.

### 2.1. Sample

The convenience sample already collected primarily in the questionnaire validation study [36] was used (*n* = 47), complemented with newly answered questionnaires (*n* = 69) from patients followed at the same reference center for the treatment of drug-refractory epilepsy, in order to achieve a representative number. A large sample is preferable for factor analysis, as it provides greater statistical power and increases the precision of parameter estimates. According to Tabachnick and Fidell (2013), the minimum recommended sample size for factor analysis is 100 cases [44]. For this study, we used the same inclusion criteria as the Keto-check validation paper [36], which were: (1) age between 0 to 19 years; (2) predominantly oral feeding; and (3) being under classic ketogenic diet treatment. Patients using exclusive gastrostomy and therapy with other types of ketogenic diet, such as the modified Atkins diet, were excluded from the study.

### 2.2. Exploratory Factor Analysis

All data were tabulated in an Excel spreadsheet and then transferred to the Factor software [45] for a parallel exploratory factor analysis [46].

Factor is a free, complete, and user-friendly program for exploratory factor analysis and semiconfirmatory analysis models. Since it was developed more than 10 years ago, it has been constantly updated with recent proposals for factor analysis [47].

The univariate descriptive results were generated by polychoric correlation, which is advised when the univariate distributions of ordinal items are asymmetric or with an excess of kurtosis. The method used to handle missing values was hot-deck multiple imputation in the exploratory factor analysis [48]. The adequacy of the polychoric correlation matrix was tested with Bartlett’s Test of Sphericity when a significant value indicates that the correlation matrix is not an identity matrix and is suitable for factor analysis [49]. Kaiser–Meyer–Olkin (KMO) was also performed, in which values above 0.6 are considered adequate for factor analysis [50].

The parallel exploratory factor analysis is based on the comparison of a matrix of data collected with a matrix of data generated by chance through artificial intelligence tools. It was established that a sample of 500 data matrices (bootstrap samples with 95% confidence intervals) would be generated, which estimated the variance matrix. The procedure for determining the number of dimensions used is according to the optimal implementation of parallel analysis [51]. The method for factor extraction was robust diagonally weighted least squares (RDWLS).

The closeness to Unidimensionality assessment was checked with 3 tests: unidimensional congruence (UniCo), which values larger than 0.95, explained common variance (ECV) with values larger than 0.85, and mean of item residual absolute loadings (MIREAL) lower than 0.300 suggests the data can be treated as essentially unidimensional [52].

### 2.3. Confirmatory Factor Analysis

The software Factor made the confirmatory analysis with the same sample immediately after the exploratory factor analysis checking the robust goodness of fit statistics.

The software performed all indexes with a bootstrap of 95% confidence interval:

- Root mean square error of approximation (RMSEA), whose values below 0.08 indicate good model fit [53];
- NCP (estimated noncentrality parameter) is a measure used in statistical power analysis for hypothesis testing. It is typically used in the context of testing for the significance of the difference between two means, such as in a *t*-test. It was adjusted with 26 degrees of freedom and the test of approximate fit considered the null hypothesis RMSEA < 0.05 [54];
- NNFI (non-normed fit index), also known as the Tucker–Lewis Index (TLI), is a goodness-of-fit index used in structural equation modeling (SEM). The NNFI is a relative fit index that compares the fit of a hypothesized model to a baseline model. It is calculated as the difference in the chi-square values of the hypothesized model and the baseline model, divided by the degrees of freedom of the hypothesized model. An NNFI value of 1 indicates perfect fit, while values closer to 0 indicate poorer fit [55];
- Comparative Fit Index (CFI): values above 0.95 indicate good model fit [56];
- GFI (goodness-of-fit index), first proposed by Jöreskog and Sörbom (1981), generally, a GFI value of 0.90 or higher is considered indicative of good fit, although this threshold may vary depending on the specific research context [57].

### 2.4. Graphical Presentation

According to all the analyses performed, the authors created a graphical presentation in order to illustrate the inter-relations between factors and items.

### 2.5. Replicability Analysis

To assess construct replicability, some analyses were made:

- Generalized H index (G-H): the H index evaluates how well the set of items represents the common factor. This value is between 0 and 1 and values greater than 0.80 suggest that the latent variable was well defined; that is, it tends to be stable across studies, while a variable with lower values tends to change across studies [52];
- Cronbach’s alpha and composite reliability: with values ranging from 0 to 1, with higher values indicating greater internal consistency. Cronbach’s alpha can be classified as when it reaches values greater than 0.8, “almost perfect”; from 0.61 to 0.8, “substantial”; from 0.41 to 0.6, “moderate”; between 0.21 and 0.4, “reasonable”; and less than 0.21, a “small” consistency is considered [58,59]. Composite reliability is a measure of internal consistency reliability that is similar to Cronbach’s alpha but is based on the concept of a latent variable model. It is calculated by taking the sum of the variances of the observed indicators and dividing it by the sum of the variances of the observed indicators plus the error variances [60];
- McDonald’s ordinal omega method: it is used when the scale items are measured on an ordinal or categorical scale. It is based on the concept of hierarchical factor models and provides an estimate of the proportion of variance in the observed scores that is due to a general underlying factor. Ordinal omega can range from 0 to 1, with higher values indicating greater internal consistency. It is recommended for use with Likert-type scales and other ordinal or categorical data [61].

## 3. Results

The questionnaire (Keto-check) is based on 10 questions of answers with options on a Likert scale of five alternatives. The sample number reached previously in the validation of the instrument was 47 patients [36], complemented by filling out another 69 questionnaires, reaching a total sample number of 116 completed questionnaires, which are described in Table 1.

A polychoric correlation matrix suitability analysis resulted in a Bartlett statistic of 1276 (with 45 degrees of freedom and *p* = 0.0001) and a Kaiser–Meyer–Olkin (KMO) test of 0.56022 (0.000–0.714 95% confidence interval).

If the analysis were based on eigenvalues, the questionnaire would be structured in three factors, as shown in Table 2. Based on this analysis, when the eigenvalue is less than one, it means that the inclusion of a new factor is not justified, since the principle of this method is the reduction of the minimum number of factors that explain the model.

**Figure 1:**
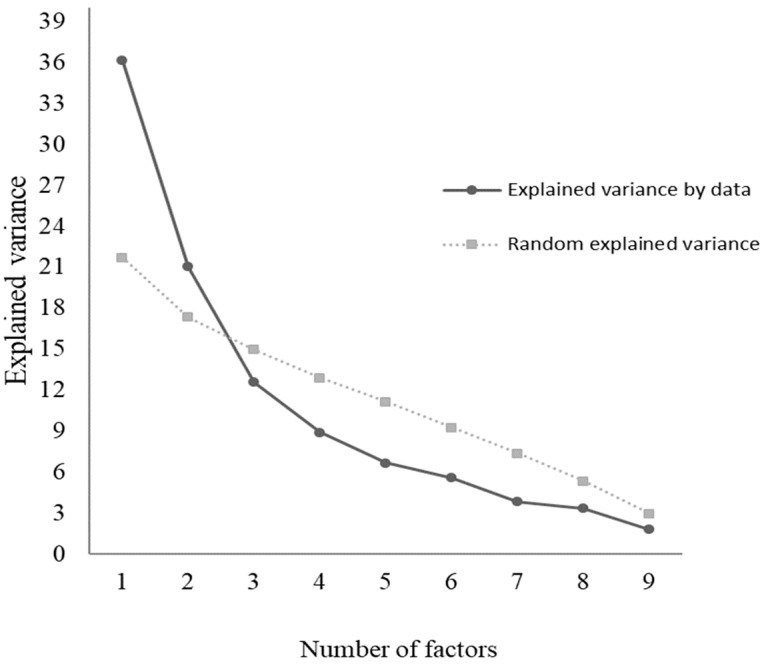
Analysis of the number of factors associated with the analyzed latent variable (adherence to the ketogenic diet), according to collected data or data randomly generated.

Table 3 demonstrates the variance based on a comparison with 500 matrices randomly generated by artificial intelligence using a raw data permutation method [51]. From the principle of this method, the variance generated by chance cannot be greater than the variance generated by the observed data. As seen in Figure 1, the number of factors must be considered before crossing the presented data. Therefore, in contrast to what is indicated by the analysis of the eigenvalues, the parallel analysis indicates the presence of only two factors.

The analysis of proximity to one dimensionality indicated the following confidence intervals: UniCo value of 0.821 (95% confidence interval 0.749–0.912); ECV 0.643 (95% confidence interval 0.524–0.749); and MIREAL of 0.341 (95% confidence interval 0.270–0.446), which indicates that the data cannot be treated as one dimensional [52].

The confirmatory factor analysis indicated an RMSEA of 0.063 (95% confidence interval = 0.0000–0.2104) considered fair, with an NCP of 7.475 (*p* = 0.791), accepting the null hypothesis of RMSEA < 0.05. The NNFI resulted in 0.872 (95% confidence interval = 0.059–1.148), CFI in 0.926 (95% confidence interval = 0.538–1.091), and GFI in 0.897 (95% confidence interval = 0.805–0.958).

The results of the factor loadings for each observed item (questions) and the respective commonality can be seen in Table 4.

**Figure 2:**
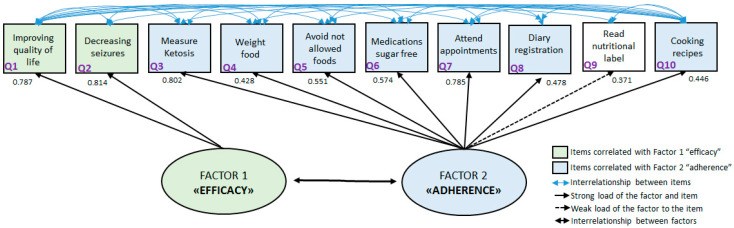
Graphical representation of the factorial analysis of the Keto-check instrument.

Figure 2 shows the schematic result of the factorial analysis, with an indication of the factorial loads for each question (item) of the questionnaire (observed items). The latent H in this study resulted in 0.896 for Factor 2, which the authors considered as “adherence” and 0.819 for Factor 1, which the authors considered as “efficacy”. The “efficacy” factor is correlated with the affirmations related to the caregiver’s perception about the reduction of crises or improvement in the quality of life. The other factor was related to all other items except reading nutritional labels (question 9) and, for that reason, was denominated “adherence”. Figure 2 also shows through arrows the linkage between the factors “efficacy” and “adherence” and the interrelationship between all items.

The Cronbach’s alpha of the questionnaire resulted in 0.793, but this evaluation does not take into account the difference between the variances of each question. McDonald’s ordinal omega method takes into account the factor loading and the importance of each item within the factor. It can be interpreted as the square of the correlation between the scale score and the latent variable common to all indicators in the infinite universe of indicators of which the scale indicators are a subset. The value in this study was 0.799. Another test used to estimate reliability that takes into account the importance of the item, through factor loadings, is the composite reliability (CR) [60], which can be seen in Table 5, with a value of 0.857.

## 4. Discussion

Evaluating adherence is challenging and increasing it in long-term interventions is even more difficult. Therefore, validating a questionnaire that reports adherence to ketogenic therapy is essential to identify areas that could improve patient care. This study confirms the validity of the Brazilian ketogenic therapies’ adherence questionnaire through a more detailed analysis. It is important to conduct a factorial analysis to determine which factors interfere with the latent variable, so as not to generate an excess of factors that could complicate the analysis unnecessarily.

The two factors that could summarize the KDTs adherence questionnaire are “efficacy”, related to the first and second items (improvement of quality of life and seizures reduction), and “adherence”, related to all the remaining items (monitoring ketosis, checking the weight of consumed food, keeping strictly to the prescribed diet, avoiding using drugs with carbohydrates, attendance in consultations, checking food labels, and cooking ketogenic recipes at home).

The first factor, “efficacy”, is reported in the literature as a very important factor that affects adherence to ketogenic therapies. Patients only continue therapy if it is effective and acceptable [62]. The other items in the questionnaire are related to how the patient or caregiver responds to the guidelines given in consultations and whether they seek to comply with the recommendations on a daily basis.

Kinsman and colleagues (1992), in a study of 58 cases of KDT for intractable seizure disorders, affirm that the patient only remains on therapy if it is effective and/or acceptable [62]. Mady and colleagues (2003), in a retrospective study of adolescents treated with KDT, also agree that in terms of compliance, efficacy is very important [63]. These findings agree with the study of Jagadish and colleagues (2019); using MAD for children with refractory epilepsy, it was reported that nearly half of the patients that discontinued the therapy did so because of a lack of efficacy [64].

The second factor found in this analysis, called by authors “adherence”, could assess all the other factors that could affect the adherence itself. This factor could cover all the needs of organization, precision, and discipline on items such as filling the monitoring instruments: food diary and ketosis, weighting food, avoiding certain foods and medications that contain carbohydrates, attending appointments, and cooking specific recipes. All these items are important in order to assess the level of compliance of the caregiver/family in the treatment.

### Strengths and Limitations

This article has some important limitations. One limitation is the use of the same database to perform exploratory and confirmatory factor analysis, which could bias the results. Another important limiting factor would be the sample number; a larger number could enhance the results.

One of the major problems found in the Keto-check questionnaire could not be resolved by this different analysis of data presented in this article. The problem is the arbitrary classification of the adherence results. Therefore, the ideal would be to compare it with data collected on ketonuria and/or ketonemia to establish a classification with ad hoc analysis. Unfortunately, there are no available data to perform this analysis, but certainly, it could be realized in future research.

The major strength of this paper could be considered the methodological approach. In the exploratory phase, with statistical robustness through the network and parallel analysis, the correlated bidimensional structure of Keto-check with at least nine items became clear. The confirmatory factorial analysis confirmed the findings of the exploratory phase, indicating good evidence of psychometric validity and the possibility of widespread use of the measure in the national context.

Damasio and colleagues (2013) [65] also used a similar methodological approach previously to describe the psychometric properties of the questionnaires. The analyses with exploratory factor analyses and confirmatory factor analyses make it possible to present a reliable and plausible factor structure in a resilience scale. A Portuguese study evaluating the adherence to antihypertensive medication through the Medication Adherence Universal Questionnaire also used confirmatory factor analysis for the development and validation of this instrument [26].

Considering medication adherence, a recent article by Krousel-Wood and colleagues (2021) [66] highlights several factors that may influence adherence to clinical treatment protocols, either positively or negatively. Four primary domains can impact the overall adherence negatively:

- Social factors: these include stress, limited practical and social support, a chaotic environment, and an unhealthy lifestyle;
- Patient-related factors: poor language skills, deficient planning and organizational abilities, emotional instability, concerns about illness, feelings of helplessness, memory deficits, depression, and negative affectivity;
- Health-system-related factors: inadequate interaction and communication between patients and healthcare professionals, perceived discrimination in the healthcare system based on race, ethnicity, education, or income, and limited access to healthcare services;
- Treatment and disease-related factors: beliefs in barriers, such as unpleasant taste or side effects, actual side effects of medication, high treatment costs, low confidence in using the therapy, and apprehensions regarding potential side effects.

On the other hand, the same four domains could facilitate improved adherence, as outlined below [66]:

- Social factors: enhanced adherence can be facilitated by an increased ability to follow norms, a positive intrafamilial relationship, strong family support, such as proximity to relatives, and the possibility of receiving adequate treatment at home;
- Patient-related factors: improved self-efficacy and self-control, a positive perception of general health, beliefs about the severity of the disease, knowledge about the purpose and effects of therapy, and recognition of disease symptoms;
- Health-system-related factors: A good relationship with healthcare professionals and improved communication, particularly in terms of receiving instructions about treatment;
- Treatment and disease-related factors: positive beliefs about treatment effectiveness, perception of the necessity for treatment, confidence in treatment safety, and recognition of the benefits of treatment.

In the same way, understanding and addressing these factors can play a crucial role in promoting dietary treatment adherence and improving the overall effectiveness of clinical treatment protocols. Schoeler and colleagues (2014) [67] assessed factors related to nonadherence to ketogenic diet treatment and identified that adherence was related to a positive perception of KDTs in families convinced about the necessity of KDTs for their children. But, on the other hand, doubts regarding the necessity of this treatment and concerns about the potential long-term effects of KDTs could negatively affect the adherence. In general, parental beliefs correlated with patients’ responses to treatment [67].

Employing validated questionnaires to measure adherence presents a straightforward, rapid, cost-effective, noninvasive, and well-accepted approach to enhance understanding of patient adherence factors that may impact the treatment.

Since adherence is an essential concept in treatment efficacy, more studies are needed to cover all these mentioned aspects of adherence. Studies in other populations could be performed to reach a wider extrapolation of the presented results.

## 5. Conclusions

Through this study, it was possible to deepen the analysis of factors involved in adherence to ketogenic therapy and confirm the validity of this tool. Adherence is the key to improving the effectiveness of treatment in ketogenic dietary therapy. Therefore, improving knowledge about this factor can lead to an improved approach to the patient, enhancing the positive outcomes of the treatment.

## Figures and Tables

**Table 1:** Descriptive analysis of the data observed in the questionnaires.

Question	Average (from 0–5)	Confidence Interval (95%)	Variance	Distortion	Kurtosis
1	3.862	(3.53–4.19)	1929	−1.055	−0.287
2	4.086	(3.77–4.40)	1734	−1.303	0.329
3	4.276	(4.02–4.53)	1.114	−1.594	1993
4	4.828	(4.66–5.00)	0.505	−4.683	21.242
5	4.362	(4.06–4.66)	1.576	−1.820	1.720
6	4.319	(4.04–4.60)	1.372	−1.812	2.079
7	4.914	(4.83–5.00)	0.130	−5.536	36.301
8	4.431	(4.16–4.70)	1.245	−2.228	3.880
9	4.836	(4.68–4.99)	0.416	−4.752	23.136
10	4.707	(4.52–4.89)	0.587	−3.277	10.945

**Table 2:** Variance explained by Eigenvalues.

Question	Eigenvalue	Variance Ratio	Cumulative Proportion of Variance
1	**3.61200**	0.36120	0.36120
2	**2.10215**	0.21022	0.57141
3	**1.26059**	0.12606	
4	0.89100	0.08910	
5	0.66426	0.06643	
6	0.55790	0.05579	
7	0.39421	0.03942	
8	0.33412	0.03341	
9	0.18375	0.01838	
10	0.00002	0.00000	

**Table 3:** Parallel analysis based on the minimum of analysis factors.

Question	% Variance Real Data	% Random Variance
1	36.2051	21.9183
2	21.0499	17.5260
3	12.5966	14.9696
4	8.9175	13.0191
5	6.6575	11.0909
6	5.5924	9.1722
7	3.8519	7.2660
8	3.3287	5.2410
9	1.8008	2.9558

**Table 4:** Matrix of factor loadings without rotation and commonality for each question of the Keto-check questionnaire.

Question	Factor 1	Factor 2	Commonality
1	**0.787**	0.379	0.762
2	**0.814**	0.298	0.752
3	0.217	**0.802**	0.690
4	−0.237	**0.428**	0.240
5	−0.112	**0.551**	0.316
6	0.218	**0.574**	0.377
7	0.068	**0.785**	0.621
8	−0.309	**0.478**	0.324
9	−0.138	0.371	0.157
10	−0.232	**0.446**	0.253

**Table 5:** Composite reliability estimated, according to Raykov’s method, 1997 [60].

Question	Factorial Loads	Error Variance	Error Squared Variance
1	0.787	0.381	0.619
2	0.814	0.337	0.663
3	0.802	0.357	0.643
4	0.428	0.817	0.183
5	0.551	0.696	0.304
6	0.574	0.671	0.329
7	0.785	0.384	0.616
8	0.478	0.772	0.228
9	0.371	0.862	0.138
10	0.446	0.801	0.199
**Composite reliability**			**0.857**

## Data Availability

All research data can be obtained by contacting the correspondence author.

## Supplementary Materials

The following supporting information can be downloaded at: https://www.mdpi.com/article/10.3390/nu15173673/s1, Table S1: Keto-check questionnaire.
