# Supplementary material for: Factor Analysis of the Brazilian Questionnaire on Adherence to Ketogenic Dietary Therapy: Keto-Check
Source: Nutrients. 2023 Aug 22;15(17):3673. doi: 10.3390/nu15173673 (PMC10489998; doi:10.3390/nu15173673)
Supplement: Supplementary file 1 [file nutrients-15-03673-s001.zip › nutrients-2569630-supplementary.pdf]

foods MDPI

Keto-check questionnaire, English version, 2022 [35]

KETO-CHECK - ASSESSMENT TOOL FOR KETOGENIC DIET ADHERENCE<br>Next you will read 10 sentences. Circle the alternative of how much you agree or disagree with the phrase in your reality and routine or with the care with your child or teenager.	
I. After the beginning of the ketogenic diet, I realize that my child / adolescent has improved the quality of life in general (considering physical health, communication, emotional, social life, positive effects on family dynamics, learning and performance in therapies).	1- Strongly disagree<br>2- Partially disagree<br>3- I do not know how to answer<br>4- Partially agree<br>5- I totally agree
II. After the beginning of the ketogenic diet, I realize that the number and / or duration and / or intensity of epileptic seizures in my child / adolescent has decreased.	1- Strongly disagree<br>2- Partially disagree<br>3- I do not know how to answer<br>4- Partially agree<br>5- I totally agree
III. After starting the ketogenic diet, I measure my child's / adolescent's ketosis (in the blood or urine) and it remains in frank ketosis (in the purple urine strip: ++ or 80mg / dL to +++ or 160 mg / dL OR in blood: 2-5 mmol).	1- Strongly disagree<br>2- Partially disagree<br>3- I do not know how to answer<br>4- Partially agree<br>5- I totally agree
IV. After starting the ketogenic diet, I weigh all the foods (raw) offered to my child / adolescent.	1- Strongly disagree<br>2- Partially disagree<br>3- I do not know how to answer<br>4- Partially agree<br>5- I totally agree
V. After the beginning of the ketogenic diet, my child / adolescent never ate food that was not prescribed by the nutritionist (there was never an escape: eating hidden or someone else offering food that is not allowed or using more or less of the ingredients).	1- Strongly disagree<br>2- Partially disagree<br>3- I do not know how to answer<br>4- Partially agree<br>5- I totally agree
VI. After the beginning of the ketogenic diet, all medications that my child / adolescent uses were exchanged for sugar-free medicines.	1- Strongly disagree<br>2- Partially disagree<br>3- I do not know how to answer<br>4- Partially agree<br>5- I totally agree
VII. I attend all appointments scheduled with a nutritionist and neurologist.	1- Strongly disagree<br>2- Partially disagree<br>3- I do not know how to answer

	4- Partially agree<br>5- I totally agree
VIII. After starting the ketogenic diet, I make notes in a notebook daily with food consumed, number of seizures, measured ketosis and complications (fever, vomiting, refusal to eat).	1- Strongly disagree<br>2- Partially disagree<br>3- I do not know how to answer<br>4- Partially agree<br>5- I totally agree
IX. After starting the ketogenic diet I always read the labels (carbohydrates, proteins, sugars) of all industrialized products and I always ask the nutritionist if it is allowed, before offering this product to my child / teenager.	1- Strongly disagree<br>2- Partially disagree<br>3- I do not know how to answer<br>4- Partially agree<br>5- I totally agree
X. I cook all my child's meals at home, I always try to vary preparations and look for new recipes.	1- Strongly disagree<br>2- Partially disagree<br>3- I do not know how to answer<br>4- Partially agree<br>5- I totally agree
TOTAL POINTS - ADD CIRCULATED NUMBERS	
· Insufficient adherence: 10 to 35 points<br>· Good adherence: 36 to 45 points<br>· Excellent adherence: 45 to 50 points	
